# Music Ensemble as a Resilient System. Managing the Unexpected through Group Interaction

**DOI:** 10.3389/fpsyg.2016.01548

**Published:** 2016-10-07

**Authors:** Donald Glowinski, Fabrizio Bracco, Carlo Chiorri, Didier Grandjean

**Affiliations:** ^1^Neuroscience of Emotion and Affective Dynamics Lab, Swiss Center for Affective Sciences, Faculty of Psychology and Educational Sciences, University of GenevaGeneva, Switzerland; ^2^Department of Science of Education, Faculty of Psychology and Educational Sciences, University of GenovaGenova, Italy

**Keywords:** resilience, music ensemble performance, social interaction, attention, anticipation, adaptation

## Abstract

The present contribution provides readers from diverse fields of psychology with a new and comprehensive model for the understanding of the characteristics of music ensembles. The model is based on a novel heuristic approach whose key construct is resilience, intended here as the ability of a system to adapt to external perturbations and anticipate future events. The paper clarifies the specificity of music ensemble as an original social and creative activity, and how some mechanisms, at an individual (cognitive) and group (coordination) level, are enacted in a particular way that endows these groups with exceptional capacity for resilience. There is now a wealth of evidence isolating the psychological mechanisms involved in these processes. However, there is much less focus on conditions in which the group has to face unexpected and potentially performance-disruptive events. The resilience approach offers a more thorough explanation of the regulatory strategies that musicians may resort to in order to maintain their performance at an optimal level. Music ensembles of different size are presented as case studies of how such systems (and their individual members) resist error and maintain joint performance. Three hypothetical scenarios are further proposed that epitomize resilient or non-resilient musical teams. The present contribution further proposes hypotheses and formulates predictions on which combinations of individual and group factors foster team resilience. This model further accommodates the most recent findings in neuroscience and experimental psychology. Besides highlighting the potential of music ensemble for psychological research, it offers hints about how resilience could be trained.

## Introduction and Plan

Recent literature suggested that music can be considered a kind of social glue, since its most commonly observed features cross-culturally relate to things that allow people to coordinate their actions. As a result, collaborative music making can provide a glimpse into the processes that bring people together and enable social bonding in groups (Turino, 2008; Savage et al., 2015). At an evolutionary level, the strength of such social bonds has been critical, especially in scenarios when the group was exposed to unpredictable and variable events that could jeopardize their safety. In modern times, such dynamics have been particularly developed in *adaptive teams* who deal with processes that are time critical and occur in real time, such as special forces (Bechky and Okhuysen, 2011), firefighter units (Feese et al., 2013), surgical room operators (Vashdi et al., 2013), or music ensembles (D’Ausilio et al., 2015). On the one hand, they all show balance between exposure to unpredictable and variable events and, on the other, reliance on procedures and rules (Amalberti, 2013; Bracco et al., 2014). At this level, the team is the main actor for the safe and effective performance of the system because the task is too complex to be managed just by individuals, or just by applying rigid stereotypical procedures, or because the task requires coordination between several units (i.e., here individuals) to be achieved. Even if competencies and overlearned skills are crucial in such groups, a critical quality of the team is also its *flexibility* in adapting the learned procedure to the challenging situation (Zellmer-Bruhn et al., 2003; Salas et al., 2015). Firefighters and doctors have life-threatening issues to deal with that are different from the unpredictable situations managed by musicians. However, from a cognitive point of view, the capacity to flexibly adapt to ongoing situations, the ability to cope with unexpected events, and the skills required to cooperate and manage the critical condition are similar for both high-risk professions and musicians. Both categories represent teams operating in time-critical and real-time conditions. In this context, resilience, a concept inherited from physics and ecology and applied to the field of industrial safety, can provide a useful framework to understand the reciprocal impact of individuals’ strategies and group behavior in order to efficiently adapt against perturbations and achieve specific goals. Resilience is strictly defined as the ability of a system to adapt to external perturbations and anticipate future events (Hollnagel et al., 2011). Here we show that this approach may be salient in its application to the study of music ensemble, since it allows predictions about how cognitive and social competencies and skills of individuals can interfere with or promote the collaborative process at stake during group performance.

In order to make clear the unique heuristic potential of the resilience approach for collaborative music making with respect to other current approaches (e.g., Rabinowitch et al., 2013; Keller, 2014), we revisited two emblematic test cases of music ensemble performance: string quartet and orchestra. The first additional value of the resilience approach is to situate the music ensemble with respect to other human group activities in terms of their characteristics along the risk exposure continuum (Amalberti, 2013). A music ensemble can be compared to one type of resilient system, one that is highly exposed to unexpected events, since it relies on team performance and is dynamically exposed to continuous perturbations both from inside and outside the group (e.g., a string quartet musician playing out of time with respect to the others, or a noisy audience). This broader view and this emphasis on risk is already a first benefit of the resilience approach, as it reveals a factor that is often implicitly considered: the thrill and the challenge faced by musicians provoked by the ever-changing situation of music performance in a group.

String quartets and orchestras represent two contrasting cornerstones of music ensembles: in string quartets, musicians, like a self-managed team, share an equal responsibility for the achievement of group performance (Gilboa and Tal-Shmotkin, 2010); in orchestras, the group of musicians is asymmetrically led by a unique conductor (Gnecco et al., 2014). These two types of organization may lead to distinct coordination strategies. Keller (2014) has provided a general framework to study music ensembles that integrates several psychological mechanisms to explain the capacity of musicians to share esthetic objectives through well-tuned body coordination and social interaction. There is now a wealth of evidence isolating the psychological mechanisms involved at the individual and group action levels. However, there is much less understanding of the processes enacted to deal with perturbations and to maintain group cohesion in a dynamic way. Focusing on timing within the music ensemble, Keller (2014) showed that the greater the horizontal deviation (e.g., timing of successive sounds), the more challenging it is to maintain optimal vertical relations (e.g., degree of synchronization). The capacity to adjust each tempo one with another in real time is extremely challenging and requires overlearned cognitive and motor skills that can be developed through intensive instrumental training. This fine-tuned collective adjustment is thought to be critical to give music its vitality and esthetic appeal. In this context, the level of perturbation considered so far by Keller is restricted to the manipulation of specific time-lag differences between sounds and to the observation of the extent to which musicians can adjust dynamically. The resilience approach can encompass a wider variety and magnitude of perturbations (e.g., including noisy audience, musician’s blackouts, musician’s mood) and can provide further details about the regulatory strategies that musicians may enact during their performance to tackle these perturbations. The empirical work by Glowinski et al. (2014) and Dardard et al. (2016) on music ensemble may complement Keller’s findings by providing behavioral markers (e.g., gaze convergence, expressive qualities of the musician’s movement) to characterize how these regulatory strategies can be enacted to foster the team’s resilient properties. Gaze convergence coupled with smooth synchronous movement of the heads were found to characterize shared effort to minimize disruptions and to promote efficient coordination among the string quartet’s musicians across the performance (Glowinski et al., 2014). Research efforts should focus on further specifying the psychological mechanisms (attention, anticipation, adaptation, etc.) that can be quantified behaviorally and falsified within the resilience framework to further establish its scientific contribution.

## Toward a Resilient Performance Model

**FIGURE 1:**
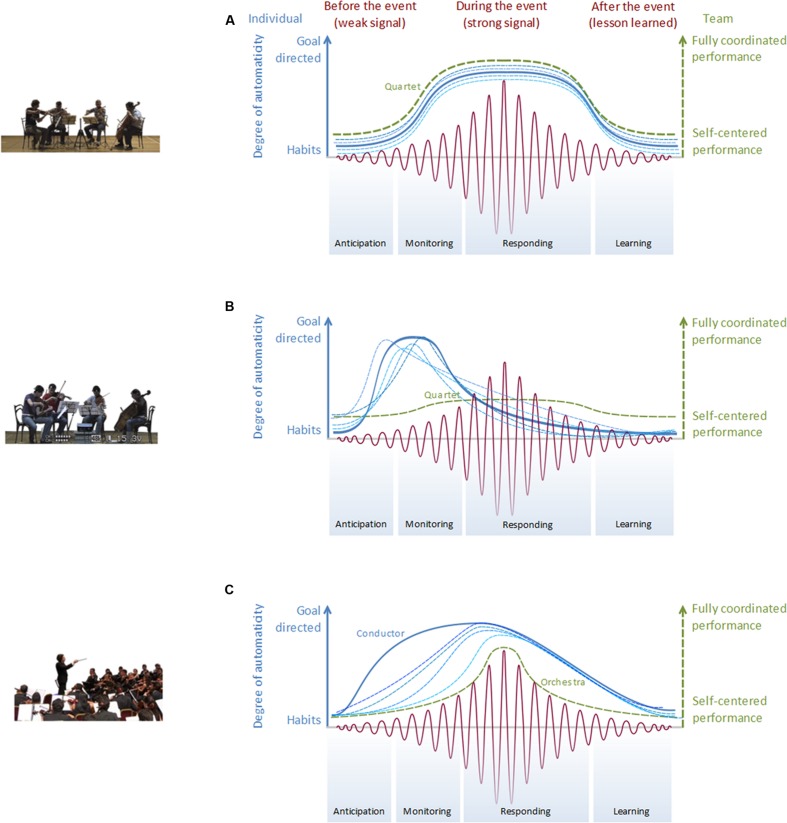
**The dimensions of the resilience approach.** Red lines represent the magnitude (amplitude) of the perturbation (weak vs. strong signal); blue lines represent the levels of cognitive effort (automatic performance vs. focused attention on the task); dotted lines represent individual musicians; full lines represent the average performance in panels **(A,B)** and the conductor in panel **(C)**; green lines represent the levels of team coordination (from self-centered to fully coordinated performance). From top to bottom, **(A,B)** represent two string quartet cases, resilient and non-resilient, respectively, and **(C)** represents the orchestra in a resilience situation. The dotted lines in panel **(C)** represent the performance of a sample of players in the orchestra. The conductor shows a higher response to the perturbation and can lead the team to perform a resilient adaptation as individuals gradually succeed in coordinating one another (see Resilient Example in the Orchestra). The magnitude of the perturbation could be very low (e.g., when the signal is weak, it is barely noticeable and its effects on performance are negligible) or high (e.g., when the signal is strong, it requires an immediate reaction to prevent performance breakdown). The cognitive effort could be low (e.g., based on habits, skilled actions, routine operations), or high (e.g., based on focused attention to the task, diagnosis of the situation, and development of a goal to cope with it). Team coordination could be low (e.g., the performance could be self-centered, with all the members engaged in their own activity), or high (e.g., the performance could be the result of team coordination, where the focus is no longer the individual action, but the adequate interplay with other team members).

We propose a model implementing the four critical features typical of a resilient system, i.e., its capacity to anticipate, monitor, respond, and learn (**Figure 1**), in order to investigate which psychological mechanisms are enacted and how they relate to one another to optimize joint performance.^1^ The focus is on risk and offsetting perturbation management. According to Hollnagel (2011), a system is resilient if it anticipates and monitors the ongoing situation, characterizing the magnitude of the internal or external perturbations and their potential impact on its operations; it responds to expected and unexpected variability, distinguishing between potential disturbances or opportunities that may either disrupt or empower the task achievement; it learns from what happened, optimizing, and capitalizing the experience in order to handle future events; and it anticipates future situations, being mindful and responsive toward changes that lie beyond the range of current operations.

These four abilities are tightly linked and should be trained as a whole in order to develop a system’s resilience. The training and development of these abilities should first focus on the optimization of individual cognitive resources (e.g., attentional processes) and team coordination skills (e.g., implicit non-verbal interaction, perspective taking) in order to monitor perturbations (Salas et al., 2015). This first step provides the system with the relevant information about the potential disruptive event, permitting to reallocate attentional resources and changing the coordination mode of operations within the team to provide a more adapted response (e.g., shifting from an autocratic to a shared leadership or *vice versa*, Guastello, 2007). When the learning is consolidated (e.g., through post-performance debriefing and sense-making activities), the system can develop the capacity to foresee and anticipate future perturbations by capitalizing upon past experiences. The enactment of these four abilities may depend on the appropriate appraisal of the perturbation at hand. Following Hollnagel et al. (2011), perturbation can be represented as a sine signal that increases its magnitude over time (red lines in **Figure 1**).

A resilient system should notice this perturbation, thanks to an adapted degree of sensitivity that is well tuned to the specific performance, and react in a proportional way to its magnitude. A suboptimal allocation of individual and team resources would lead to either over- or underreaction to a potential threat or problem (Pavlidis et al., 2012). An optimal allocation of resources implies distinguishing between weak (e.g., the subtle body-expressive features characterizing a bored audience) and strong signals (e.g., a musician’s blackout during the performance) and provides an adequate response. This distinction does not aim at classifying a perturbation dichotomously (i.e., good vs. bad), but distinguishing between gradual differences in intensity that may affect the performance. A perturbation could hence become the cue for a new and creative course of action, as happens in jazz music. As stated by Pezzulo et al. (2013), positive perturbations can become opportunities if they are shaped as *signals*, i.e., “parameterizable deviations from the action’s optimal trajectory so that the signaling action retains its pragmatic goal” (p. 2). Therefore, the deviation may bring novelty and open new paths, without compromising the global mission of the team.

Human cognitive systems are able to learn and progressively automatize chunked series of actions related to specific contextual information and then give rise to so-called specific habits. In this context of human interactions, habits are not defined only by the coupling of sensory and motor actions: they refer to a more complex coupling and patterning between percepts, representations, inferences, and actions in which specific dynamic percepts integrate and merge with related specific inferences, coupled with fine-tuned motor actions. We claim that such complex dynamic patterning and its potential expected perturbations can be trained to form habits, allowing performers to cope with perturbations and to then be resilient.

## Model Features

The model proposed in this paper is schematically depicted in **Figure 1**. It aims at offering a synthetic view of the three dimensions that can impact upon the system’s performance. These dimensions include (i) the magnitude of the perturbation, (ii) the levels of cognitive efforts, and (iii) the levels of team coordination. For each combination of these three dimensions, one can predict whether the system is resilient or not, i.e., whether at each moment of the perturbation, it enacts one of the four critical features of a resilient system. With respect to a concert condition, where all musicians aim at performing their best, studies may thus devise original experimental conditions to create perturbations that often resume to create boundary conditions to which musicians learn to react, so that their learning and expertise can be revealed (e.g., Badino et al., 2014; Gnecco et al., 2014). Drawing upon recent published studies (e.g., Glowinski et al., 2014; Dardard et al., 2016), we briefly consider three test cases from string quartets and orchestras that illustrate two resilient systems (**Figures 1A,C**) and a non-resilient system (**Figure 1B**).

### Resilient Example in the String Quartet

Individual cognitive processes and team coordination dynamics underlying the string quartet performance can be revisited according to the resilience approach as follows: at the cognitive level, team members can begin their operations by playing as usual (see the left vertical axis and the related blue curves in **Figure 1A**). At the beginning, when their cognitive effort is low, they perform by means of overlearned and quasi-automated processes because they do not detect any perturbation in their work domain (e.g., they all perfectly know the music piece from their repertoire). When the perturbation signal increases its magnitude, they can monitor the presence of unwanted variability and change their cognitive effort accordingly (e.g., unexpected pace acceleration of the rhythm provoked by the first violinist). The other musicians notice the perturbation and they are focused on finding a way to cope with it, reallocating their cognitive resources and changing their way of playing and interacting with each other (e.g., adapting the pace to abrupt acceleration, adjusting the timing, trying to alert the first musician to be more responsive to their music proposals). When the perturbation is under control and the signal is dampened, their successful coping strategy will become part of the team’s experience, and their cognitive effort can decrease again to the normal, quasi-automated performance (e.g., go back to the refrain on which they agree). The same dynamics can be described according to the interaction among the musicians (the green right axis and the related green dotted line in **Figure 1A**). Team coordination and flexibility is low for usual and routine operations, while it increases as soon as the perturbation occurs and each musician is forced to purposefully coordinate with teammates in order to face the unexpected event. As team attention is focused on coordination, the members need to put some effort into finding a creative and flexible way to cope with the perturbation. This phase is based on non-verbal sharing of information, bodily coordination, and mutual gazes to direct attention toward relevant sources of information (Badino et al., 2014; Glowinski et al., 2014). Eventually, when the perturbation has been successfully coped with, each team member can return to the performance, decrease effortful coordination, and focus on the execution of their own part.

### Non-resilient Example in the String Quartet

The resilience model can also be useful in describing non-resilient performances, as depicted in **Figure 1B**. Here, the ineffective performance is not due to the low effort of musicians, but to the lack of team coordination. As soon as they notice a possible perturbation (e.g., the unexpected variation of the first violinist), their effort increases, but they focus on their individual performance and stick to the technical aspects of the performance. As a consequence, their increased cognitive effort can easily turn into, e.g., emotional strain and distress, which can lead, in a vicious circle, to an even greater narrowing of attention on the task (the blue lines in **Figure 1B**). Unfortunately, this effort does not correspond with team coordination, which remains scarce and inadequate to cope with the strength of the signal (the green dotted line vs. the red one in **Figure 1B**). The team is not resilient, it overreacts to the signal in an uncoordinated way, and effort rapidly drops, possibly because of, e.g., frustration and anxiety while the perturbation is still high.

### Resilient Example in the Orchestra

The last test case revolves around the conductor-orchestra relationship. The resilience framework may enhance understandings of the process through which the conductor manages the continuous attentional shift of musicians to ensure a robust group performance (Wöllner and Halpern, 2016, on attentional flexibility). **Figure 1C** specifically illustrates how individual cognitive processes and team coordination dynamics interact in a resilient system. The full blue line represents the conductor’s effortful and focused attention on the perturbation: such focused attention is already elicited when the signal is weak; it remains high as long as the perturbation signal increases its magnitude (e.g., some of the musicians are out of time and tune). The conductor may notice that the team is not aware of the incoming perturbation, or that some musicians are not ready to cope with it (e.g., they do not manage to keep their tempo regular with the others). She therefore shifts her resources to enhance team performance and coordination in order to successfully cope with the signal. She becomes the pivot of the whole task and helps the group to move from a collective performance of single musicians to a coordinated, flexible team (see the dotted green line in **Figure 1C**). According to this model, a resilient team will face an unexpected event with a coordinated increase in attentional resources, moving from automated performance to focused attention on the new problem. This process will correspond to a shift in, e.g., the locus of control of the coping strategy (Keller, 2014) from individuals to the team by means of a non-verbal, flexible interaction among teammates, facilitated by the conductor. Gnecco et al. (2014) demonstrated that musicians’ degree of gaze convergence can reveal how successful the orchestra director is in attracting musicians’ attention and ensuring coordination among them.

## Implications for Future Research

These examples illustrate the potential of the resilience approach as a heuristic framework, not only in music ensembles, but, more generally, in groups of interacting individuals, as it provides hypotheses on how individual cognitive skills and group coordination properties can be combined to handle perturbations efficiently. It also suggests new lines of research aimed at uncovering, e.g., the underlying neural and perceptual processes that allow for individual and groups to be resilient. These aspects are currently under investigation, specifically by tapping into the recent contributions on perceptual decision making under conditions of uncertainty (Summerfield and de Lange, 2014) or on the role of neural substrates, which are key for optimizing behavior and shaping habits and progressive automatization of procedures through experiences (e.g., Graybiel and Grafton, 2015). We refer in particular to how the expectation developed through specific motor training expertise in the field of music can interfere with the predictive capacity of others’ actions (Blakemore and Decety, 2001) and the efficient achievement of joint actions (Sebanz and Knoblich, 2009; Frith and Frith, 2012). Such a research framework, which integrates motor theory, simulation theory, and predictive coding, may help refine the results obtained so far (Novembre and Keller, 2014) and could further contribute to the attempt at formalization of music interaction (Cross, 2013, for example). In addition, the prediction by the resilient approach that an individual within a group, able to smoothly shift from automated to focused attention and goal-directed tasks, will handle perturbations more efficiently could gain from the concept of affective flexibility (Hollnagel, 2011), i.e., how emotional processes may interfere or facilitate one’s ability to change from one cognitive state to another. The details of behavioral processes and information flow between musicians could further benefit from advances in social signal processing, in consideration of the behavioral features that can be extracted at individual (e.g., motion activity) and group levels (e.g., level of synchronization; Eagle and Pentland, 2006). In this context, this resilience framework allows an understanding of team interactions of variable degrees of complexity more systematically and in a *scalable* way. Questions still remain unanswered; for example, how the resilience framework could benefit from research on group agentivity, on the sense of togetherness (Rabinowitch et al., 2013), or on the impact of personality factors upon team coordination (Schmid et al., 2009).

We claim that this framework can provide an original and incisive perspective on known cases typical of music ensembles, which can also have implications for the development of novel strategies for training musicians. Specifically, it can help in the understanding of concepts that are commonly used in the field of creative and social activities, such as intuition or creativity (Seddon and Biasutti, 2009), which are loosely defined or about which it is difficult to have clear agreement. This framework also provides a conceptual apparatus to better analyze and understand how improvisation practice can help not only musicians, but also individuals in other organizational contexts, to deal more flexibly with perturbations and maintain internal cohesion within the team.

## Author Contributions

All authors have made a substantial, direct, intellectual contribution to the work. DGL and FB contributed to the conception and together with CC and DGR implement the conceptual framework.

## Conflict of Interest Statement

The authors declare that the research was conducted in the absence of any commercial or financial relationships that could be construed as a potential conflict of interest.

## Footnotes

1 The model depicted in **Figure 1** has been conceptually described in Bracco et al. (2014) as representing the cognitive dynamics of a resilient performance. It is originally framed as a cycle, starting from routine activities and performed by skilled professionals who are almost *isolated* in their task. As soon as they notice a weak signal, their cognitive effort increases and they share the information at the team level, moving from an individual to a group coordinated performance. This condition cannot last for long, since it is cognitively demanding. The team will look for a way to cope with the signal (a new procedure, an insight, a makeshift solution), which will be progressively embedded in their experience as a new routine.
